# Acceptability of Home‐Based Urine Self‐Collection for Cervical Cancer Screening Among Women Receiving Care at the Arab Community Center for Economic and Social Services in Michigan

**DOI:** 10.1002/cam4.70714

**Published:** 2025-02-26

**Authors:** Timothy C. Guetterman, Christelle El Khoury, Madiha Tariq, Ghada Aziz, Asraa Alhawli, Martha L. Alves, Elizabeth Haro, Emma A. Butcher, Alexandra H. Vinson, Diane M. Harper

**Affiliations:** ^1^ Department of Family Medicine University of Michigan Ann Arbor Michigan USA; ^2^ Oakland County Michigan Dearborn Michigan USA; ^3^ Arab Community Center for Economic and Social Services (ACCESS) Dearborn Michigan USA; ^4^ Department of Physical Medicine and Rehabilitation University of Michigan Ann Arbor Michigan USA; ^5^ Department of Learning Health Sciences University of Michigan Ann Arbor Michigan USA; ^6^ Department of Obstetrics and Gynecology University of Michigan Ann Arbor Michigan USA; ^7^ Department of Women's and Gender Studies University of Michigan Ann Arbor Michigan USA

**Keywords:** cervical cancer screening, Middle Eastern‐North African (MENA), qualitative study, self‐sampling

## Abstract

**Background:**

Michigan's Middle Eastern‐North African (MENA) community is an essential and growing part of the state's population. However, MENA individuals are underrepresented in the research literature due to a lack of recognized demographic categorization. Prior work shows that MENA women face barriers to traditional clinician‐directed cervical cancer screening. This study aims to capture the perspectives of MENA women about home‐based urine cervical cancer screening using HPV kits and to assess whether such methods could positively impact future screening intent.

**Methods:**

Through collaboration with a community partner in southeast Michigan, we recruited MENA women ages 30–65, with 44 completing the study. Participants used urine HPV self‐sampling kits at home and then shared their perspectives through a phone interview. We used an inductive, thematic approach to analyze the interviews, which captured experiences with home‐based self‐sampling, screening preferences, and impact on future screening intent.

**Results:**

Participants found that urine home‐based self‐sampling was acceptable as a convenient and comfortable way to screen for cervical cancer. Most (80%) preferred self‐sampling over traditional clinician‐directed screening and preferred collecting urine samples at home (73%) rather than in the clinic. Overall, 80% reported that access to urine self‐sampling would positively impact their future screening intent.

**Conclusions:**

MENA participants in this study positively received home‐based cervical cancer screening using urine HPV self‐sampling kits. These findings support the clinical implementation of self‐sampling and home‐based cervical cancer screening to increase participation, particularly among those in under‐screened communities.

## 1 Introduction

Michigan has the second largest population of Arabic‐speaking ancestry in the US [1]. The population is increasing yearly, with newly settled immigrants making up 10.5% of Michigan's Arab American community [2]. Many self‐identify as MENA (Middle Eastern‐North African) but are largely unseen in research data because there is no MENA race/ethnicity option on the US Census [3]. Their resulting categorization, usually under white ethnic categories, hinders our understanding of MENA health barriers and facilitators. This misalignment is most apparent when studying population health concerns such as cervical cancer prevention.

Cervical cancer screening has evolved over the past century, from glass slides for conventional cytology to primary HPV testing with genotyping for high‐risk types [4, 5, 6]. While national guidelines recommend primary HPV testing from clinician‐directed sampling, studies have shown the accuracy and validity of self‐sampling to provide equivalent HPV detection [7]. Self‐sampling is an acceptable screening alternative for under‐screened communities [8].

Clinician‐directed screening involves a speculum‐based exam, which presents barriers for MENA women who screen less frequently for cervical cancer than other demographic groups [9, 10, 11, 12, 13]. Previous studies of MENA women indicated that culture, religion, and having a provider of the same gender were facilitators of cervical cancer screening [14]. However, more recent work indicates that MENA women, many of whom were born in other countries, no longer worry about the culture or religion of their healthcare provider [15].

Our work aimed to capture the perspectives of MENA women in Michigan about home‐based HPV self‐collected urine cervical cancer screening across different ages of the screening spectrum. The results can further inform whether such methods could positively impact future screening among this unique population.

## 2 Methods

### 2.1 Participants

We recruited women in partnership with a community‐based organization serving a large Arab American community (Arab Community Center for Economic and Social Services, ACCESS) in southeast Michigan. Screening eligible patients was identified at their routine visit to invite them to the study. Sequential enrollment occurred from June 2020 to February 2021 until we reached at least 20 women aged 30–45 and 46–65. We allowed for snowball sampling, which resulted in participants from Michigan (30), Pennsylvania (11), Kentucky (2), and New York (1). For eligibility criteria, see Table 1. The study was approved by a large university medical center's institutional review board (IRB) and by internal review by the community partner. Participants received $50 compensation.

**TABLE 1:** Participant eligibility.

Inclusion criteria	Exclusion criteria
Self‐identifying as MENA	Pessary use for pelvic floor support or bladder control
Age 30–65 years	Self‐reported pregnancy or planning to become pregnant within the next 6 months
Having a cervix (no hysterectomy)	Current cancer diagnosis (not including non‐melanoma skin cancer)
Due or overdue for cervical cancer screening (no screening within the last 3 years)	Self‐reported poor health status

### 2.2 Home‐Based Screening

Those interested and eligible to participate were provided written informed consent and received a urine sampling device, instructions, and a demographic survey by mail. The women were not told anything about the accuracy of urine‐based HPV collection prior to their participation. The urine collection device was Colli‐Pee (Novosanis, https://novosanis.com). Materials were available in English and Arabic, and participants were encouraged to contact the study team with questions at any time. Device instructions are in Appendix A.

### 2.3 Quantitative Survey

We used the National Cancer Institute (NCI) Health Interview National Trends Survey (HINTS) stem questions from the cervical cancer module [16], a community‐based cross‐sectional health survey developed for the MENA population [17] and specific questions about screening experiences gathered from prior work [18] for each participant to answer after using the self‐screening device, mailing the completed survey and specimen to us upon completion.

### 2.4 Semi‐Structured Qualitative Interview

After using the kit, one of two bilingual (Arabic/English) team members contacted each participant for a telephone interview within a week after kit use. About two‐thirds of the interviews were conducted in English (66%), with the remaining (34%) in Arabic. Questions assessed participants' experiences with home‐based urine screening, their preferences for self‐sampling versus clinician‐directed screening, their preferences for self‐sampling locations (home or clinic), and its potential impact on future screening intent. The research team transcribed the English interviews, and the Arabic‐speaking research team transcribed the Arabic interviews for those who consented to an audio recording (66%). The remaining (34%) allowed the interviewer to take detailed notes, and all Arabic content was translated into English before analysis.

### 2.5 Analysis

Demographic descriptors were analyzed using frequencies, means, and standard deviations. Comparator statistics with chi‐square and Fisher's exact tests compared the perceived characteristics of urine and speculum‐based screening. Interviews were analyzed using a thematic text analysis approach [19]. First, three researchers reviewed interview transcripts to identify potential codes through an open‐coding process. Then, all met to discuss and determine a final coding framework. Two researchers independently coded each interview and compared for agreement through a consensus process. To generate themes, the whole team described relationships between codes to develop themes and discussed discrepancies for consensus.

## 3 Results

### 3.1 Demographics

A total of 44 women participated in the study: 20 aged 30–45 years and 24 aged 46–65 years (Table 2). Most were not employed (64%), had adequate income (72%), self‐reported good health (52%), and had a routine health exam in the past year (52%) (*p* < 0.05). Over a quarter (27%) had a high school education or less, significantly less than a higher education (*p* < 0.05). Table 3 shows that a majority (71%) reported having had a speculum exam, prior pregnancy (86%), and having given birth (82%). Around a third (30%) reported currently being menopausal. The two age groups differed significantly by income (getting by or better 50% younger vs. 79% older, *p* = 0.03), history of a physical exam in the past year (30% younger, 71% older, *p* = 0.008), and being menopausal (5% younger vs. 50% older, *p* = 0.001). All 44 participants self‐identified as MENA during recruitment. More detailed items on the questionnaire resulted in 34 describing their race as MENA, seven as non‐Hispanic white, one as multiracial (Asian & MENA), and one as ‘other’ (wrote in ‘*Arab*’). Most (93%) identified as Arab and 7% as Chaldean.

**TABLE 2:** Population descriptors from the quantitative survey.

Race/ethnicity	*N*	%
Asian	1	2.3
MENA^a^	34	79.1
Hispanic	1	2.3
Arab	7	16.3
Location		
Rural	1	2.3
Small town	2	4.5
Mid‐size town	17	38.6
Suburbs	7	15.9
Large town or city	17	38.6
Education		
Less than 8th grade	5	11.9
8–11th grade	4	9.5
High school graduate or GED	8	19.0
Vocational/technical school	2	4.8
Some college	5	11.9
College graduate	16	38.1
Graduate school	2	4.8
Employment		
Full time	10	22.7
Part‐time	6	13.6
Unemployed for less than 1 year	4	9.1
Unemployed for more than 1 year	6	13.6
Homemaker/Caretaker	16	36.4
Disabled	2	4.5
Income		
Living comfortably	8	18.6
Getting by	20	46.5
Finding it difficult to get by	11	25.6
Finding it very difficult to get by	4	9.3
Insurance status		
Employer‐based	3	7.7
Purchased on own	1	2.6
Medicaid	21	53.8
Medicare	2	5.1
None	12	30.8
Health status		
Excellent	4	9.1
Very good	6	13.6
Good	23	52.3
Fair	11	25.0

^a^ MENA means Middle Eastern‐North African ethnicity.

**TABLE 3:** Reproductive health history of the population.

How long since your last routine health checkup		
Within past year	23	53.5
1–2 years	6	14.0
3–5 years	7	16.3
More than 5 years	2	4.7
Never had a routine health checkup	2	4.7
Experienced a speculum‐based pelvic exam		
Yes	31	70.5
No	9	20.5
Unsure	4	9.1
Gravidity		
Yes	38	86.4
Parity (Mean 2.6, SD 1.1)		
0	2	5.3
1–2	13	34.3
Three or more	23	60.5
Miscarriage (Mean 0.7, SD 0.8)		
0	9	31.0
One or more	20	69.0
Abortion (Mean 0.2, SD 0.5)		
0	19	76.0
One or more	6	24.0
Reproductive surgeries		
Yes^a^	2	4.5
No	42	95.5
Current birth control		
Male condom	2	4.5
Cervical cap	1	2.3
Oral combined contraceptives	2	4.5
Copper IUD	2	4.5
Mirena IUD	2	4.5
None	35	79.5
Menopause		
Yes	13	31.0
No	27	64.3
Unsure	2	4.8
Menopausal therapies		
Hormonal replacement	40	90.9
Non‐hormonal replacement	2	4.5
None	2	4.5

^a^ One oophorectomy, one bilateral salpingo‐oophorectomy.

### 3.2 Quantitative Survey

**FIGURE 1:**
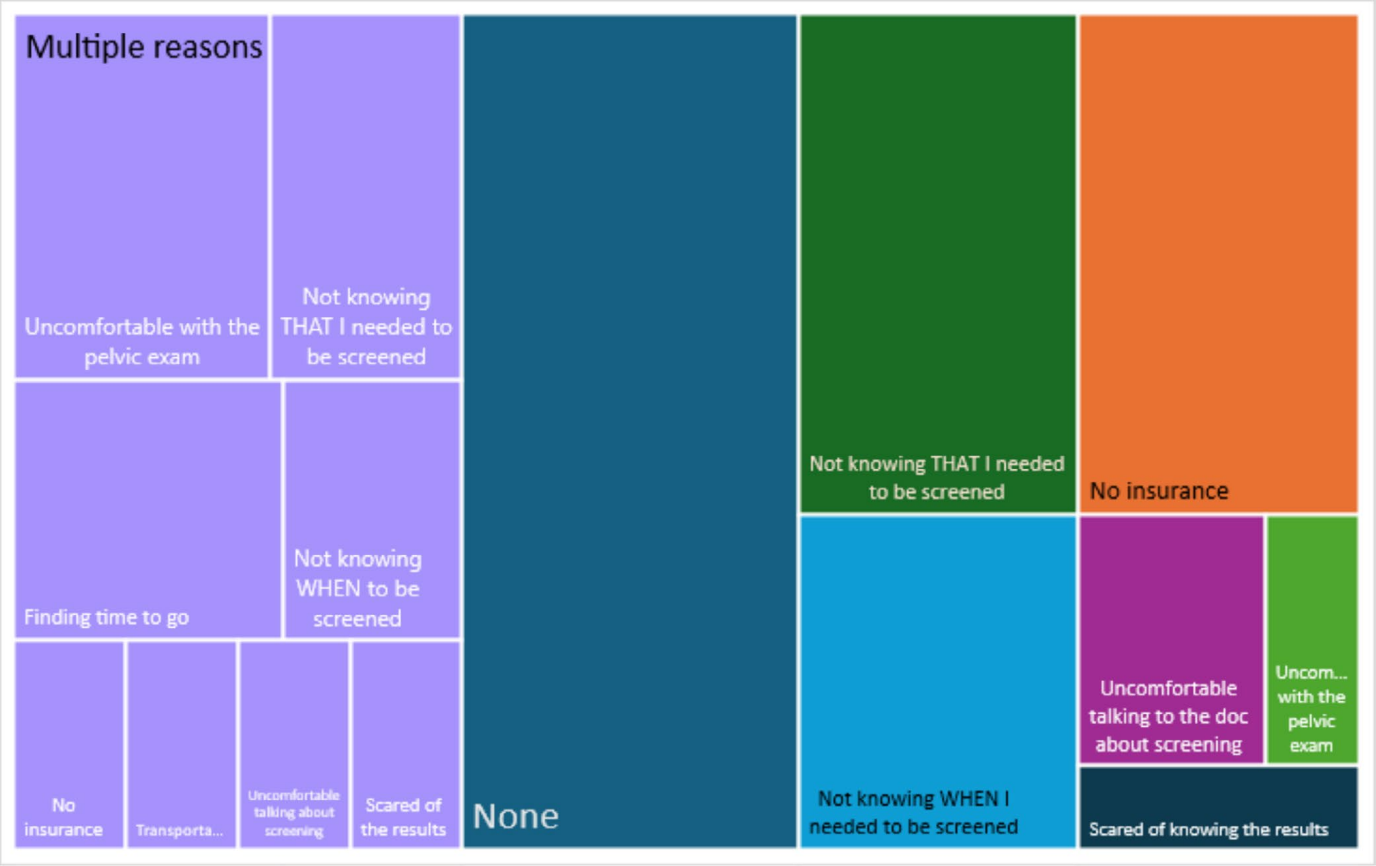
Barriers to cervical cancer screening: None, one, many. Central Dark Blue block: No barriers existed for 33% (13/39) of participants. Right multi‐colored block: Single barriers occurring in 41% (16/39) of participants are individually colored by the frequency of their identification. Left‐purple block: 15% (6/39) of participants had more than one barrier, which was proportionately identified according to its frequency of identification.

The answers to the quantitative survey described different frequencies of barriers to cervical cancer screening. One‐third had no barriers, 41% had a single barrier, and 15% had multiple barriers, as proportionately diagrammed in Figure 1. Nearly a quarter of women (23%) did not have the basic knowledge they needed to be screened.

The instructions for the urine device were easy to understand: 85.7% of women read the instructions and found them very helpful, and the rest found the instructions somewhat helpful or did not answer. Likewise, women ranked the ease and comfort of use of the urine device as 1.4 (1.7) and 1.8 (2.3), respectively, on a 0–10 Likert scale where 0 was very easy/comfortable and 10 was very hard/uncomfortable.

**FIGURE 2:**
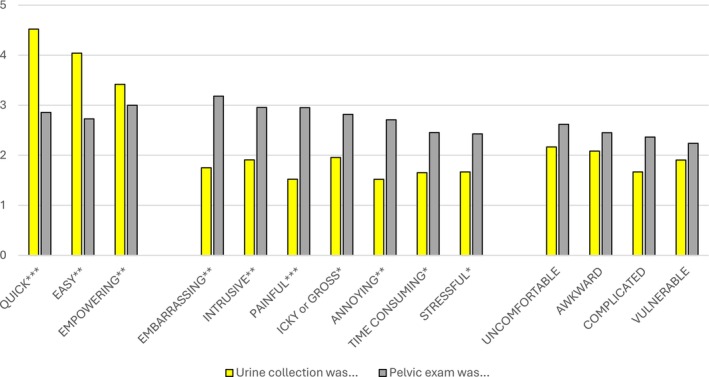
Mean rankings of attributes of the urine collection and pelvic exam techniques. The first three attributes are positive, and the last 11 attributes are negative. Rankings: 1 = strongly disagree, 2 = disagree, 3 = neither agree nor disagree, 4 = agree, 5 = strongly agree. All comparisons are corrected for multiple comparisons. *p < 0.05, **p < 0.01, ***p < 0.001.

We present the Likert scale agreement scores from the women who had a speculum exam experience in the past compared to the experience of the urine collection device in the study (Figure 2). Women ranked the attributes of empowering, uncomfortable, awkward, and vulnerable the same for both the urine device and the speculum‐based exam. Women ranked the urine device significantly quicker and easier than the speculum exam and ranked the speculum exam significantly more embarrassing, painful, annoying, intrusive, stressful, complicated, and time‐consuming than the urine device.

### 3.3 Qualitative Interview

Three major themes from the analysis were that home‐based screening is convenient and comfortable, self‐sampling and screening at home are preferable when given adequate instructions, and self‐sampling kits improve the likelihood of future cervical cancer screening. The interviews ranged from 4 min 5 s to 9 min 30 s. Below, we describe each theme with illustrative quotes. Participant names were changed to maintain anonymity while reporting results.

#### 3.3.1 Home‐Based Screening Is Convenient and Comfortable

In describing their overall experience with the study, convenience and comfort were both mentioned by most participants as benefits of home‐based screening (Table 4). Participants described the time involved in going to the doctor and the ease of home‐based screening. Difficulties such as time, coordination of schedules, and transportation access can be mitigated through home‐based screening. Maryam, a 51‐year‐old participant, explained, “If someone says they are not comfortable in going to the doctor, they don't know how to drive, or they don't have time, I would say this is a great idea doing it at home…for me it's the time more than anything.” Home‐based screening was also seen as a way to avoid barriers to traditional screening appointments. The sentiment was echoed by many, who also described the ability to fit screening into their own schedule. Participants also said that because not everyone drives, home‐screening obviates the need for transportation.

**TABLE 4:** Qualitative interview: Benefits of home‐based urine screening.

	Full study cohort (*N* = 44) 30–65 years	Younger cohort (*n* = 20) 30–45 years	Older cohort (*n* = 24) 46–65 years
Convenience			
Mention frequency	32 (73%)	13 (65%)	19 (79%)
“It was easier and more convenient” (Salwa, age 55). “This is good for people who don't have time” (Mona, age 42). “The convenience of doing it at home at your own pace is more comfortable” (Nadine, age 34). “It's easy to use at home and doesn't take time” (Yasmin, age 34).			

Comfort			
Mention frequency	28 (64%)	12 (60%)	16 (67%)
“This is easier, more comfortable, and it's more private. There were no drawbacks” (Dina, age 57). “The comfort of being at home… you are not exposed” (Maryam, age 51). “It is easy, not intrusive” (Laila, age 41).			

In addition to the convenience of home‐based screening, comfort was a salient benefit for many. This factor often arose when participants compared their experience with self‐sampling to traditional clinician‐directed screening. For example, Salwa, age 55, said of Pap smears: “The procedure itself, I hate it. I haven't had the procedure for more than three years now.” Participants expressed concerns about physical comfort, indicating the kits are easier than a Pap smear, which can be painful. Iesha, age 43, added, “When you use the kits by yourself, you would know to be gentle on yourself.” Others described benefits, including emotional comfort and privacy in self‐sampling. Some participants described previous expresses with Pap smears as stressful and anxiety‐provoking. For example, Yasmin, age 34, explained, “The Pap smear is stressful, and it feels a little bit embarrassing.” Participants also explained that home‐based screening is a better experience because of more privacy, such as not having to disrobe in a clinic.

#### 3.3.2 Self‐Sampling and Screening at Home Is Preferable When Given Adequate Instructions

Participants also described screening preferences between self‐sampling and clinician‐directed sampling (Table 5). Among participants, 80% reported that they preferred self‐collection. For example, Leyla, age 42, said, “I prefer doing it myself. Some women might not understand or trust that they are doing it the right way so might want to choose doing it by the doctor. However, if the woman knows how to use the kit, she would definitely use it by herself.” Participants often explained that clear instructions for collecting the self‐sample are needed for the best experience.

**TABLE 5:** Qualitative interview. Screening preferences.

	Full study cohort (*N* = 44) 30–65 years	Younger cohort (*n* = 20) 30–45 years	Older cohort (*n* = 24) 46–65 years
Sampling preference			
Urine self‐sampling kit	35 (80%)	17 (85%)	18 (75%)
Clinician‐directed sample	4 (9%)	1 (5%)	3 (13%)
No preference	3 (7%)	1 (5%)	2 (8%)
Unclear	2 (4%)	1 (5%)	1 (4%)
“I would choose using these kits” (Yasmin, age 34). “I would prefer this one than getting the regular Pap smear. If this kit has the same result, I would prefer doing it at home” (Fatima, age 58).			

Location preference			
Home	32 (73%)	15 (75%)	17 (71%)
Clinic	7 (16%)	2 (10%)	5 (21%)
No preference	4 (9%)	3 (15%)	1 (4%)
Unclear	1 (2%)	0	1 (4%)
“I prefer doing it at home. It is a good thing to do it at home yourself, very easy, nice” (Mona, age 42). “I would rather do it at home” (Dina, age 57).			

In contrast, 9% of participants preferred traditional clinician‐directed screening. Some cited perceived aspects of care that a doctor can provide during an in‐person cervical cancer screening visit, such as examining fibroids, usually done by ultrasound surveillance. Others explained a general preference for having clinician expertise and felt more assured of the accuracy of a clinician‐directed sample. Amira, age 47, said, “To be more accurate, I would rather the doctor do it. I don't know if this is 100% accurate.” Of the remaining, three participants (7%) said they did not prefer either method, and two were unclear.

Participants also reflected on the location of self‐sampling, at home, as in this study, or during an appointment with their healthcare provider. Most participants (73%) expressed a preference for self‐sampling at home. On the other hand, 16% of participants preferred self‐sampling in the clinic setting. The reasons for preferring the clinic mostly centered on the benefit of having staff available to answer questions. This reassurance was true for Aya, age 51, who responded, “I would do it alone at the doctor's office. In case I needed anything, they could be right there.” Another reason for preferring the clinic was access to a new self‐sampling kit if there was a problem with the one just used. Finally, four participants (9%) responded that they did not have a location preference, and one was unclear.

#### 3.3.3 Self‐Sampling Kits Improve the Likelihood of Future Cervical Cancer Screening

Lastly, during the interview, we asked participants to consider how having the choice to use these kits impacted their decision or ability for future screening for cervical cancer. In response, 80% indicated that access to self‐sampling would increase their intent for future screening. For example, Laila, age 41, said, “I will be all for it, I will do it.” These positive responses were generally seen across age groups, with 90% of those 30–45 years old and 71% of those 46–65 years old saying self‐sampling would positively impact screening intent.

About one‐third of participants (36%) discussed the overall benefits of self‐sampling to promote health. Many participants were health conscious and appreciated screening for illnesses before symptoms were present. For example, Amani, age 43, explained, “Everyone should check on their health and make sure there is no hidden disease.” Self‐sampling at home was seen as a facilitator for screening and a way to promote health by taking ownership of one's own health through self‐checking. Maya, age 44, offered that she would tell her family and friends: “Do this test and make sure you're healthy.”

For others, the availability of self‐sampling would not change future screening intent. This included Lara, age 51, who stated, “It would not impact me since I am always following up with my doctor about all the screening needed.” Among these individuals, they preferred to continue to see their doctor as their primary way to maintain health.

## 4 Discussion

Overall, the MENA women in our study had a positive experience with urine home‐based cervical cancer screening using mailed HPV self‐sampling kits. Such an approach was seen as a convenient and comfortable way to screen for cervical cancer and an important tool to facilitate health. Other findings indicate that MENA culture and religion were not significant barriers to cervical cancer screening [10]. This work showed that the screening method's comfort and convenience were the most salient factors for both age groups, with convenience being essential for those in the older age group.

The high proportion of MENA women voicing a preference for both self‐sampling (80%) and home‐based (73%) screening was also similar to previous findings [20]. A large majority (80%) of our participants, especially those in the younger age group, reported that having access to such screening methods would increase their likelihood of participating in future cervical cancer screening. However, these findings indicate that some women still prefer traditional clinician‐directed screening. Allowing multiple options to complete screening might be a patient‐centered development that could increase screening attendance and follow‐up.

Education about the accuracy of these different screening methods is essential. A positive suggestion is to have written instructions with phone numbers or portal links to reach the healthcare office if there is any problem with using the device. With at‐home self‐sampling comes the possibility of uncoupling cervical cancer screening from the in‐person clinician visit and speculum exam. At this time, the science of urine HPV testing is not sufficiently developed to create a clinically accurate tool [21], as opposed to vaginal swabs, which are FDA‐approved for self‐screening [22]. Nonetheless, education for clinicians, healthcare staff, and patients must be updated for maximal understanding and process uptake [23, 24, 25, 26]. The healthcare community needs to promote the process of screening oneself for cervical cancer as a simple home test you bring to the lab, allowing the physician to use the visit to discuss the implications of the test results.

### 4.1 Strengths and Limitations

Our study was a large qualitative study of 44 MENA women across the screening age range (30–65 years) who completed focused interviews in their preferred language. With the help of our bilingual researcher staff and community partner, we elicited women's voices from an understudied community.

A limitation was that our participants self‐selected to participate in the study, meaning that those with existing biases towards traditional screening methods may have been more interested in participating and had more positive feedback about self‐sampling. In addition, recruitment occurred during the COVID‐19 pandemic, which could have positively skewed responses to home‐based screening. Lastly, MENA communities in the US are diverse by nature and include newly arriving immigrants and multi‐generation residents. However, we did not collect information on factors such as country of origin or length of residency because we are aware of the documented fears around immigration status [27]. Additionally, while we intended to capture the perspectives of Michigan's MENA community, snowball sampling expanded recruitment to include residents of the northeast (New York and Pennsylvania) and the south (Kentucky). Due to the small sample sizes of these other groups, comparisons between states could not be meaningfully assessed. Data on immigration factors and a more balanced geographical distribution would provide critical details about the diversity within the larger MENA‐American community.

## 5 Conclusion

Our results suggest that home‐based cervical cancer screening using urine HPV self‐sampling kits could increase screening uptake in MENA communities. This approach would be a convenient, comfortable, and health‐facilitating alternative to traditional clinician‐directed screening.

## Author Contributions

Conceptualization: D.M.H., T.C.G., A.H.V. Methodology: D.M.H., T.C.G., A.H.V. Validation: T.C.G., A.H.V. Formal Analysis: T.C.G., C.E.K. Investigation: C.E.K., M.T., G.A., A.A., M.L.A., E.H., E.A.B. Writing – original draft: D.M.H., T.C.G. Writing – review and editing, D.M.H., T.C.G., A.H.V., C.E.K., M.T., G.A., A.A., M.L.A., E.H., E.A.B. Supervision: E.H., C.E.K., G.A., A.A., M.T. Project Administration: E.H. Funding acquisition: D.M.H.

## Ethics Statement

University of Michigan IRBMED HUM00163301.

## Conflicts of Interest

The authors declare no conflicts of interest.

## Data Availability

Patient interviews are confidential, and hence, beyond the statements presented in the paper, it will not be available.
